# Introgressive Hybridization and the Evolution of Lake-Adapted Catostomid Fishes

**DOI:** 10.1371/journal.pone.0149884

**Published:** 2016-03-09

**Authors:** Thomas E. Dowling, Douglas F. Markle, Greg J. Tranah, Evan W. Carson, David W. Wagman, Bernard P. May

**Affiliations:** 1 School of Life Sciences, Arizona State University, Tempe, Arizona, United States of America; 2 Department of Fisheries and Wildlife, Oregon State University, Corvallis, Oregon, United States of America; 3 Department of Animal Science, University of California Davis, Davis, California, United States of America; Aristotle University of Thessaloniki, GREECE

## Abstract

Hybridization has been identified as a significant factor in the evolution of plants as groups of interbreeding species retain their phenotypic integrity despite gene exchange among forms. Recent studies have identified similar interactions in animals; however, the role of hybridization in the evolution of animals has been contested. Here we examine patterns of gene flow among four species of catostomid fishes from the Klamath and Rogue rivers using molecular and morphological traits. *Catostomus rimiculus* from the Rogue and Klamath basins represent a monophyletic group for nuclear and morphological traits; however, the Klamath form shares mtDNA lineages with other Klamath Basin species (*C*. *snyderi*, *Chasmistes brevirostris*, *Deltistes luxatus*). Within other Klamath Basin taxa, *D*. *luxatus* was largely fixed for alternate nuclear alleles relative to *C*. *rimiculus*, while *Ch*. *brevirostris* and *C*. *snyderi* exhibited a mixture of these alleles. *Deltistes luxatus* was the only Klamath Basin species that exhibited consistent covariation of nuclear and mitochondrial traits and was the primary source of mismatched mtDNA in *Ch*. *brevirostris* and *C*. *snyderi*, suggesting asymmetrical introgression into the latter species. In Upper Klamath Lake, *D*. *luxatus* spawning was more likely to overlap spatially and temporally with *C*. *snyderi* and *Ch*. *brevirostris* than either of those two with each other. The latter two species could not be distinguished with any molecular markers but were morphologically diagnosable in Upper Klamath Lake, where they were largely spatially and temporally segregated during spawning. We examine parallel evolution and syngameon hypotheses and conclude that observed patterns are most easily explained by introgressive hybridization among Klamath Basin catostomids.

## Introduction

While hybridization has generally been viewed as a force that erodes biodiversity, several authors [1–4] have hypothesized that effects of introgression are not always negative, and that introgressive hybridization can be a creative factor during the evolutionary process [5]. This perspective has been especially common in the botanical literature, and plant biologists have noted that there are groups of interbreeding species (termed syngameons) that maintain their ecological, morphological, genetic and evolutionary consistency in spite of extensive hybridization [5,6].

Introgressive hybridization significantly confounds our ability to recover accurate phylogenetic relationships [7]. Specifically, introgression results in discordant patterns of variation among loci and traits, with incongruence depending upon the adaptive significance of specific variants [8,9]. Neutral markers could flow freely across species boundaries and frequencies of different alleles would be influenced by genetic drift and biased introgression. Markers and traits under selection would be influenced by local processes, resulting in rapid fixation or loss of variants in specific adaptive and detrimental backgrounds, respectively. Therefore, traits under selection can persist in the face of strong gene flow [10], resulting in a mosaic of introgressed and locally adapted genetic variants within genomes of various species.

Alternatively, apparent discordance of characters could also arise from sympatric speciation, driven by divergent selection at specific localities. While allopatric speciation has become the null model for speciation theory [11], sympatric speciation has gained increased acceptance, and recent debate has shifted to frequency of occurrence [12]. Sympatric models have a strong local component, where positive selection potentially generates parallel evolution of different morphotypes across geographic locations. This process has been hypothesized to be important in several groups of fishes [13]; however, genomic data has indicated that this mode of speciation may not be as common in fishes as once thought and systems that were once thought to have evolved through sympatric speciation appear to reflect diversity from multiple colonization events [14].

Seehausen [2] hypothesized that some of the diversity in African Rift Lake cichlids resulted from introgressive hybridization. These species have undergone cycles of allopatric speciation, created by uplift and wet-dry cycles, followed by periods of sympatry [15]. Reproductive isolation in this group is often driven by sexual selection on male coloration and could have been disrupted by turbidity. During times of increased turbidity, increased hybridization would lead to the generation of hybrid swarms. As environmental conditions changed (e.g., water cleared), sexual selection would become significant again, leading to the observed radiation [2]. Trophic polymorphisms, which are usually the basis for adaptive speciation [16–18], seem especially common in lake fishes and can lead to rapid speciation (<15,000 yr [19]). Rapid, parallel evolution of morphology, such as reduced body plates in freshwater *Gasterosteus aculeatus*, can also result from selection on alleles that are rare in ancestral forms [20].

The key difference among introgressive hybridization and parallel evolution could be identified by examining patterns of covariation across markers. Under a model of parallel evolution, local pairs share a common evolutionary history; therefore, one would expect them to exhibit covariation of independent neutral traits, yielding correlations among all characters and geography. If patterns result from introgression among forms that evolved in allopatry, some neutral characters would be correlated with geography (with the level depending upon the amount of gene flow) due to gene exchange among forms while others would reflect past history of divergence accumulated during isolation (especially if they are maintained by selection). Therefore, if hybridization has been important, there would be an association among traits and geography for some characters, with the remainder discordant with geography, reflecting past isolation and divergence.

Fishes within the family Catostomidae (e.g., suckers) are exceptionally well-suited for assessing the potential role of introgressive hybridization in evolution. Hybridization among members is common [7, 21–23], raising the possibility that gene exchange could influence patterns of variation and play a significant role in the evolutionary process. In addition, all members of the family are tetraploid, thought to have originated through hybridization more than 50 million years ago [24]. As in many plants, duplicate copies of genes allows for adaptive genomic diversification [25, 26], creating opportunities to acquire adaptive variants that would be unavailable in most other systems involving introgressive hybridization. These combined characteristics make systems of catostomin hybridization especially valuable for investigating a broad spectrum of potential influences of introgression in animal evolution.

**Fig 1:**
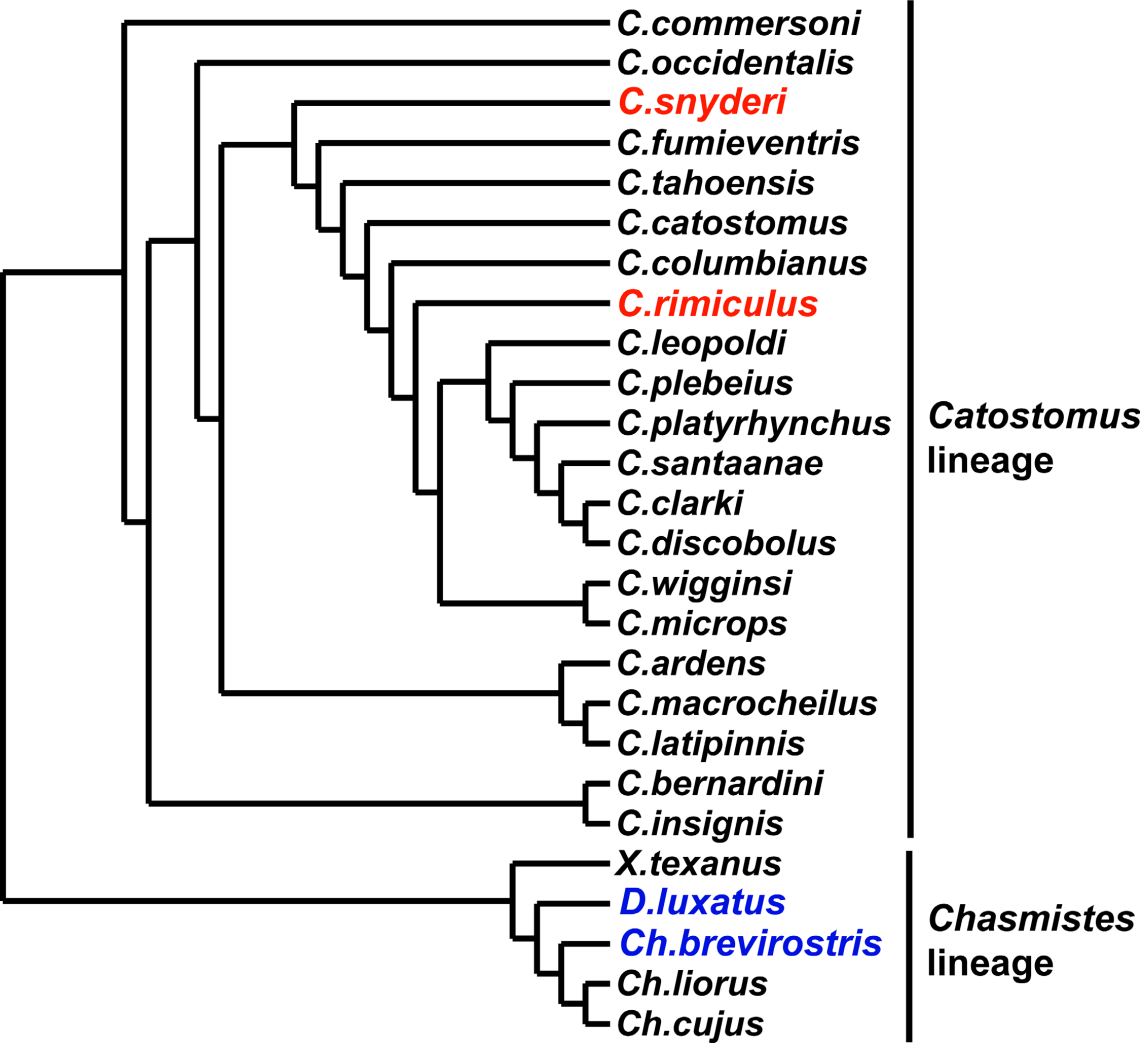
Phylogenetic relationships of catostomin suckers based on morphological characters (redrawn from [27]). *Catostomus* and *Chasmistes* lineage taxa of interest are highlighted in red and blue, respectively.

**Fig 2:**
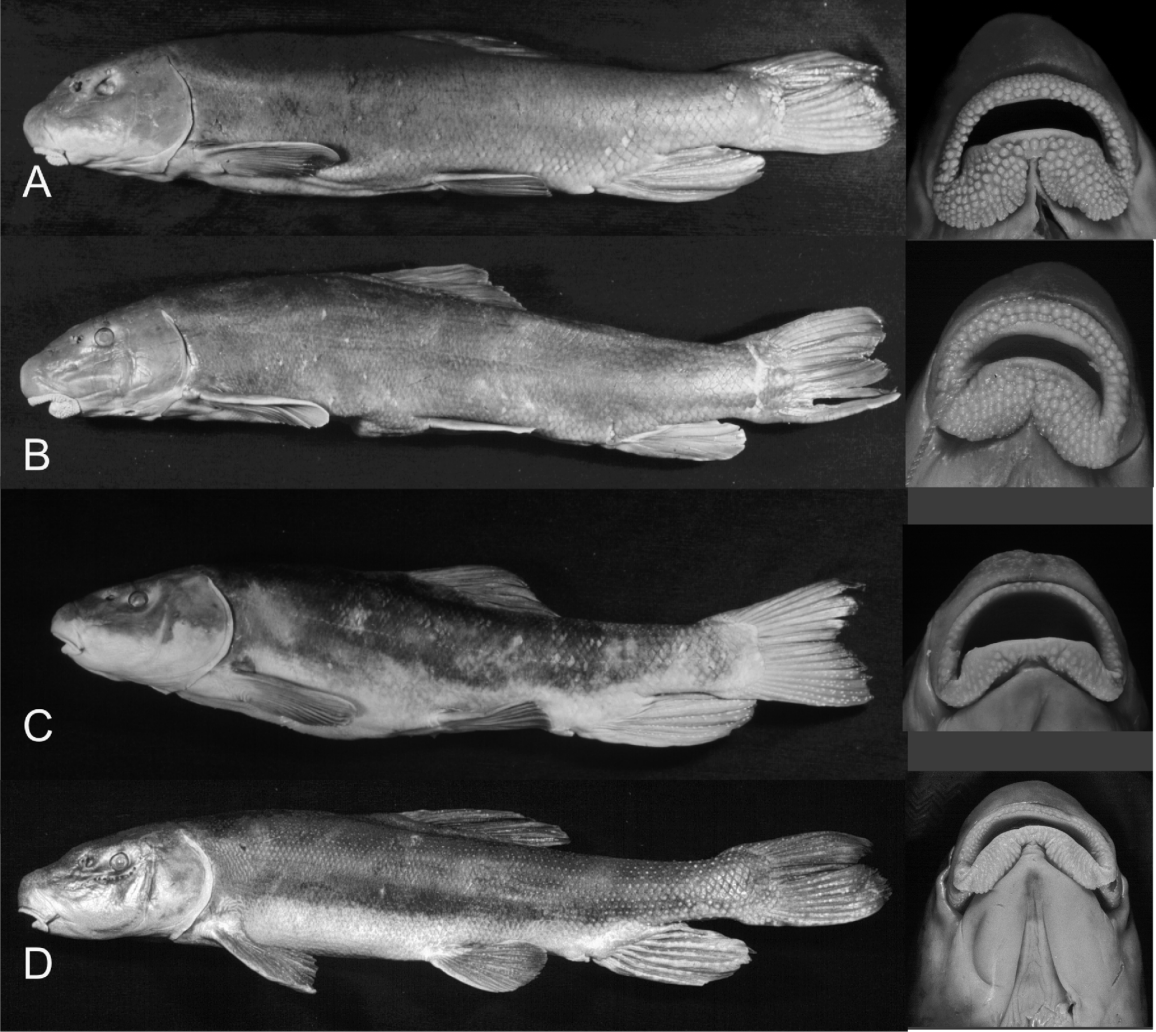
Left lateral view and ventral view of lips of representative individuals of Klamath Basin suckers. (A) *Catostomus snyderi*, OS 15893, (B) *Catostomus rimiculus*, OS 15909 (body) and OS 15906 (lips), (C) *Chasmistes brevirostris*, OS 15953, and (D) *Deltistes luxatus*, OS 15924. Body images reproduced from [31].

Smith [27] used morphological and meristic characters to reconstruct phylogenetic relationships among species in the subfamily Catostominae, identifying two major phylogenetic lineages (Fig 1). Members of the *Chasmistes* lineage (including *Chasmistes*, *Deltistes*, and *Xyrauchen*) are found in large bodies of water in western North America. These species are typified by morphological modifications that are presumably adaptations for successful persistence in these habitats. Species of the *Chasmistes* lineage also possess trophic modifications (reduction in lip fleshiness, terminal mouths, and numerous modified gill rakers) that are thought to be adaptations for pelagic lake feeding (Fig 2). This lineage is represented by five extant taxa, with fossil representatives tracing back 9 million years [7, 28–30]. The other lineage (*Catostomus*, Fig 1) is more speciose (26 species), with its members lacking such trophic modifications (Fig 2). It is widely distributed throughout streams and rivers of North America, though the majority of diversity is concentrated in the western United States and northern Mexico.

**Fig 3:**
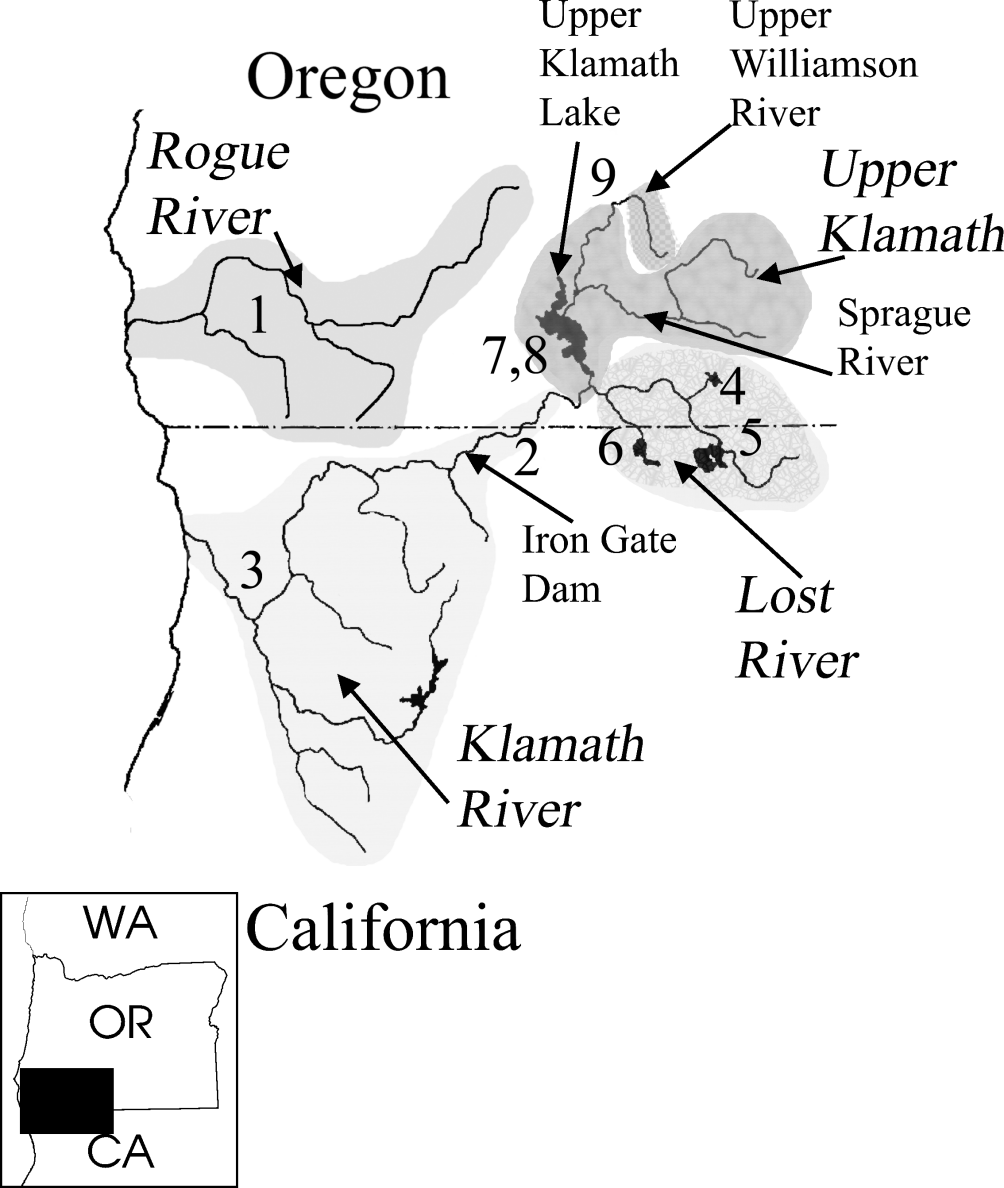
Map showing Klamath and Rogue basins with shaded sub-basins and sub-basin designations used in study. Upper Klamath sub-basin includes Upper Klamath Lake, lower Williamson River and Sprague River. Insert shows relation of basins to western states. Numbers refer to locations discussed throughout the text: 1 –Rogue R., 2 –lower Klamath reservoirs, 3– lower Klamath R. below reservoirs, 4 –Lost R. Gerber Reservoir, 5 –Lost R. Clear Lake, 6 –Lost R. Tule Lake, 7– Upper Klamath Lake, 8 –Upper Klamath Lake springs, 9 –Upper Klamath Williamson R.

The situation in the Klamath River Basin of the northwestern United States (Fig 3) makes it well suited for the study of hybridization in these fishes. This basin has a diverse catostomin fauna, represented by three genera and four species (Fig 2), with three of the species endemic to this basin. The two representatives of the *Catostomus* lineage are primarily stream forms: *C*. *rimiculus* Gilbert & Snyder 1898 (Klamath Smallscale Sucker) is found allopatrically in the Rogue River and parapatrically with the other species in the Klamath River, mainly below Upper Klamath Lake (only one specimen is known from the lake); and *C*. *snyderi* Gilbert 1898 (Klamath Largescale Sucker) occurs mostly in streams above Upper Klamath Lake and in the Lost River sub-basin (occasional specimens are taken downstream in Klamath River), including a parapatric population in the Upper Williamson River above Klamath Marsh. Two members of the *Chasmistes* lineage, *Chasmistes brevirostris* Cope 1879 (Shortnose Sucker) and *Deltistes luxatus* Cope 1879 (Lost River Sucker), are primarily found sympatrically in Upper Klamath Lake and in the Lost River sub-basin, with occasional specimens in downstream Klamath River reservoirs. Phenotypically intermediate individuals between all species in the basin have been observed [27, 28, 31], making identifications difficult, especially for some species in certain geographic areas (e.g., *Ch*. *brevirostris* and *C*. *snyderi* in Gerber Reservoir in the Lost River sub-basin [31]). In addition, levels of microsatellite divergence among these four species are comparable to those normally associated with intraspecific populations [32].

We present results from a multi-institutional collaborative study initiated to assess the patterns of genetic variation within and among species of Klamath Basin suckers. In the following, we conduct a joint population and phylogenetic analysis of anonymous nuclear DNA, mitochondrial DNA, and morphological data to further examine the evolutionary dynamics of the Klamath sucker complex. Characterization of patterns of molecular and morphological variation within species and direct contrasts of variation among these four species allows for assessment of the extent and direction of introgression and the role of hybridization in the evolution of this morphologically diverse complex of fishes.

## Materials and Methods

### Ethics Statement

Specimens were collected by personnel of the US Fish and Wildlife Service, US Bureau of Reclamation, Klamath Tribes, and US Geological Survey under state and federal permits from U. S. Fish and Wildlife Service (1992–1993 samples: Regional Blanket Permit PRT-702631 and Federal Fish and Wildlife Service Native Endangered Species Recovery Permits Number KIMBC-4, KLATRB-5, and BUETM; 2001 samples: Federal Fish and Wildlife Service Native Endangered Species Recovery Permit Number TE007907-2), Oregon Department of Fish and Wildlife Permit OR 01–095; 2002 and 2006 samples: California Department of Fish and Wildlife permit SC3849.

Tissue samples (muscle, fin, liver, heart, eye, and brain) were obtained from fish euthanized by overdose with MS-222. Carcasses, data and tissue samples were delivered to the Oregon State University Ichthyological Collection by USFWS. Tissues were also obtained by removing a piece of pectoral fin (< 3 mm square) after which fish were immediately released unharmed. This process was fast (a few seconds), required minimal handling, and caused no harm to the fish so no anesthesia was applied.

### Study Area

Specimens were collected by using a variety of nets and electroshockers from the Klamath and Rogue river basins in south central Oregon and northern California (Fig 3), a region that encompasses the entire distribution of all four species. Attempts were made to obtain samples from suspected spawning groups in spring and early summer, 1993–1994, and 2001 but 202 (51%) were collected outside the spawning seasons, August to November 1993 and 2001. An additional 21 specimens were collected from the Lower Klamath Basin in 2001–2006 where only *C*. *rimiculus* occurs.

In some cases specimens from certain areas, such as Gerber Reservoir, could be reasonably assigned to the Gerber spawning group regardless of collection date. Klamath Basin collections (Fig 3) are discussed relative to four sub-basins, with approximate coordinates for most samples in parentheses: 1) Upper Williamson—Williamson River above Klamath Marsh (42.5 N, 121.3W); 2) Upper Klamath—Upper Klamath Lake (42.2 N, 121.6 W), lower Williamson River (42.3 N, 121.5W), Sprague River (42.3 N, 121.5 W), and Link River downstream of Klamath Falls (42.2 N, 121. 8 W); 3) Lost River—Clear Lake (41.5 N, 121.1 W) and Gerber Reservoir (42.1 N, 121.1 W); and 4) Klamath River–including downstream reservoirs (J. C. Boyle (42.1 N, 122.0 W) and Copco (41.6 N, 122.2 W). Rogue River collections were from about 42.3 N, 123.3 W. Exact locality information for each voucher is available at http://www.fishnet2.net/search.aspx?c=OS.

### Morphological identification

Samples were collected during four years (see S1 Table for localities and all data). In 1993–94, 333 adult specimens were sacrificed for studies of morphological variation and used to identify diagnostic traits used for field identification [31]. Voucher carcasses from 328 specimens were deposited in the Oregon State University Ichthyological Collection (OS 15892–15915, 15917–15920, 15922–15931, 15933, 15936–15941, 15943–15944, 15946–15950, 15952–15954, 15956, 15957, 15959–15969, 17476, 17478–17480, 17487, 17490–17491) and tissues were obtained from 329 individuals for nuclear and mtDNA analyses. Opercles were removed for ageing from a subset of the voucher carcasses and ages determined by G. G. Scoppettone (US Geological Survey).

In 2001, 315 non-lethal tissue samples were collected from Upper Klamath sub-basin for additional mtDNA analyses. Specimens were identified to species in the field by experienced field biologists with the Klamath Tribes and USGS, using lower lip size relative to maxillary length, presence or absence of lower lip gap, relative snout and head length, and body shape as described by Markle et al. [31]. These were mostly *C*. *snyderi* and *D*. *luxatus* and account for the larger number of mtDNA samples for these taxa; however, 13 specimens appeared to be morphological intermediates based on conflicting characters states as determined by the field biologists (10 with a presumed parent from *D*. *luxatus* and three with presumed parents *C*. *snyderi* and *Ch*. *brevirostris*). An *a priori* hybrid category could not be consistently and objectively defined from morphology in the field, but field crews made a subjective assignment of the dominant parent; therefore, all individuals were initially provided field identification to the species they most closely resembled. This approach is conservative because misidentification would reduce our ability to diagnose forms. Because of small sample sizes for *C*. *rimiculus*, fin clips from an additional 21 specimens were obtained in 2001, 2002 and 2006, using methods described above. These samples were collected in the Lower Klamath Basin where *C*. *rimiculus* is the only species present.

### Mitochondrial DNA

Whole genomic DNA was extracted from muscle or fin tissue of 665 individuals by the proteinase-K/phenol chloroform method as modified by Tibbets & Dowling [33]. Sequence variation within and among populations and species was surveyed for two mitochondrial genes, subunits 2 and 4L of NADH dehydrogenase (ND2, ND4L), using analysis of single-stranded conformational polymorphisms (SSCPs [34]) and genotypes are provided in S1 Table. These fragments were selected because of their length, availability of primers, and rates of evolution. Primers for ND4L (ARG-BL [35], ND4L_RBS_, 5’TGTTGGAAATAGCATAATCG3’) allowed for characterization of the entire gene, whereas those for ND2 (ND2-F1, 5’ATCTCATCCCCTCGCTACCA3’ and 585R 5’GGGTTAGTTGAGGGGCATAC3’) amplified a 298 bp fragment in the middle of the gene. Fragments were amplified through 20 or 30 cycles (for ND2 and ND4L, respectively) of 94°C, 1 min, 48°C, 1 min, and 72°C, 2 min.

A single representative of each SSCP variant from each gel was then amplified using polymerase chain reaction (PCR) and sequenced on a 377 ABI prism automated sequencer as described in Dowling et al. [34], allowing for assessment of consistency of scoring across gels and characterization of sequence variation. All sequences obtained were aligned by eye, using MacDNAsis (Hitachi Software Engineering Company, Tokyo, Japan). Composite haplotypes were generated for each individual by designating each specific mobility variant with a unique letter and number for each ND4L and ND2 SSCP fragment, respectively. Haplotypes were designated in order of discovery; therefore, the labeling system does not reflect relationship among variants.

Because of the large number of samples, selected individuals representing each major haplotype group were sequenced for the entire cytochrome *b* gene (primers LA, HD, and HA reported in Dowling & Naylor [36] and LD_RBS_ [5’ ACCCTAACACGATTCTTTGC 3’]) and the remainder of ND2 (B2 [5’ CTCCTGGTGCTTCCTCTACA 3’] and E_GILA_ [37]), providing a total of 2499 characters for additional phylogenetic context. Fragments were amplified through 25 cycles of 94 C, 1 min, 48 C, 1 min, and 72 C, 2 min. Samples of catostomin suckers (including 11 additional taxa) representing the diversity within the tribe [27] were used to provide phylogenetic context, and the tree was rooted with *Erimyzon sucetta*, *Minytrema melanops*, and *Moxostoma erythurum*. Representatives of SSCP haplotypes and all sequences for phylogenetic analysis were deposited in GenBank (Accession numbers KU697909-KU698077).

### Nuclear markers

A subset of the above individuals was screened for variation at several nuclear markers and genotypes are provided in S1 Table. Nuclear markers were identified using anonymous fragment length polymorphisms (AFLPs) and sequence analysis of anonymous clones. DNA for analysis of AFLPs was extracted from fin and muscle samples, using the TNES-Urea and standard phenol-chloroform procedure of White & Densmore [38] as modified by Asahida et al. [39]. Characterization of AFLPs was used as a tool to prescreen a subset of individuals (five representatives from two populations for each species) to identify taxon-specific nucleotide polymorphisms [40]. In total, 64 AFLP-primer combinations [41] were used to screen for taxon-specific variants. Sequence variants were aligned to reveal sequence polymorphisms between species and analyzed for presence of restriction sites.

It is important to point out that the morphological identifications used by Tranah and May [32] were the initial field identifications, often based on geography and not the morphological identifications used by Markle et al [31] or herein. Using this approach, three codominant, taxon-specific markers were identified and primers developed for each of the diagnostic characters (Cri1, Cri2, Csn1). Primers were generated and used to screen samples collected in 1993–1994, using several different methods. Locus Cri1 was a single nucleotide polymorphism (SNP) (GenBank AF335378 and AF335379) that produced a diagnostic, codominant *Tsp*45I restriction site, and individuals were screened by digestion of amplification products and electrophoresis. Locus Cri2 is a deletion that produced two alleles differing in fragment size (GenBank AF335380 and AF335381), and individuals were genotyped by PCR and electrophoresis. Locus Csn1 displayed a SNP (GenBank AF335382 and AF335383) that did not contain a diagnostic restriction site; therefore, individuals were screened by SSCP.

In the second approach, anonymous nuclear loci were isolated from a pUC18 genomic library constructed from DNA extracted from *Ch*. *brevirostris* muscle tissue (OS 015963-B) [42–44]. Total DNA (10 ug) was digested with Sau3AI (Promega, React 4), size fractionated on a 0.8% agarose gel, and the fragments of 300–600 bp purified and ligated into the *Bam*HI site of pUC18. From assays of 202 recombinant clones 28 low copy number clones were sequenced using pUC18 primers, and primers designed using Oligo Version 4.0 (National Biosciences Inc., Plymouth MN [44, 45]).

One individual of each upper basin species (*D*. *luxatus* OS 015922, *Ch*. *brevirostris* OS 015963-B, *C*. *snyderi* OS 015900-F) was surveyed for sequence differences at 28 loci, using optimized PCR amplification, reflecting a total of 10,421 bp. PCR products were sequenced in both directions and aligned using SeqEd (version 1.0.1, Applied Biosystems Inc.). Surveys of variant clones used either denaturing polyacrylamide gel electrophoresis (PAGE [46]) or SSCP analysis as described above. One informative locus (L4) was identified, exhibiting two codominant alleles (Genbank AF362135 and AF362136) that differed in size (A = 397 bp, B = 386 bp).

### Statistical analyses

Phylogenetic analyses of sequences from SSCP fragments of mtDNA sequences were completed using maximum parsimony algorithms as implemented in PAUP* (vers. 4.0b10, D. L. Swofford, Sinauer Associates, Inc., Sunderland, MA, 2001). Unrooted most-parsimonious trees were obtained through heuristic search (TBR method, simple addition). Full sequences of the three mtDNA genes were analyzed by parsimony and maximum likelihood (GTR+I+G model identified by the Akaike information criterion as determined by ModelTest 3.07 [47]. For parsimony analysis, trees were recovered by heuristic search, using the TBR method with 10 random addition sequences. Bootstrap analysis was performed using the fastsearch option with 1000 and 100 pseudo-replicates, respectively. Contrasts among tree topologies (morphological [Fig 1] and best ML tree) for the mtDNA sequence data were performed as described in Felsenstein [48], where two times the difference in ML scores fits a chi square distribution with one degree of freedom.

Where appropriate, Hardy-Weinberg equilibrium was tested for nuclear loci, using Arlequin, version 3.5 [49]. We also used Arlequin to quantify levels of genetic variation among all samples (*F*_*ST*_) as well as partitioning variance into among species (*F*_*CT*_) and among samples within species (*F*_*SC*_) components, testing for significant differences at these levels. Neighbor-joining was used to cluster sample populations by pairwise estimates of *F*_*ST*_ with MEGA6 [50]. Confidence in nodes for the nuclear gene topology was assessed by bootstrapping the nuclear data (1000 replicates) and constructing a neighbor joining tree, using POPTREE2 [51]. Tests of cytonuclear disequilibrium were performed for locus Csn1 in the Williamson River sample, using the program CND [52].

To examine the relationship among character sets, we conducted an ordination of the genetic data with and without diagnostic morphological characters [31], using nonmetric multidimensional scaling (NMS) as implemented in PC-ORD [53]. NMS has been used to describe nonhierarchical geographic structure in genetic and morphological data because it does not assume linearity, uses ranked distances, and can accommodate any distance measure or relativization [54–56]. Unlike Discriminant Function Analysis, ordination of individuals is done without reference to their assigned group membership, although diagnostic characters were included. Only individuals with complete data were included in this analysis (N = 252). Multivariate outliers were identified as individuals whose total Euclidean distance was greater than 2 standard deviations from the mean and removed from final analysis, as their influence on descriptive axes can be large [53]. Multistate categorical variables were converted into binary characters by presence (1)/absence (0) coding as recommended by McCune and Grace [56]. The number of converted characters coded as present for each character was N-1, with the remaining state represented by absence for all characters. For example, the four mtDNA clades were represented by three binary characters, with the LUX haplotype lineage coded as 1,0,0; RIM1 coded as 0,1,0; BR coded as 0,0,1; and RIM2 coded as 0,0,0. The other genetic and morphological traits were converted into binary characters in a similar manner, yielding the following (number of characters in parentheses): L4 (2), Cri1(2), Cri2(2), Csn1(1). There were two morphological characters converted into binary characters: gap in lower lip (present, absent, deformed– 2 characters) and posterior extent of lower lip relative to end of maxillary (anterior, equidistant, posterior– 2 characters). Quantitative meristic and morphometric characters were used as is, including number of gill rakers, number of post-Weberian vertebrae, number of lateral line scales, snout length as a proportion of head depth, and length of the contact of lower lips as a proportion of eye diameter. All character states were normalized by the standard deviation (also called Z scores). We used squared Euclidean distance, began each run of 400 iterations with six axes (*k* = 6) and a random starting configuration, set the instability criterion at 0.00001, and ran 40 runs with real data and 50 runs with randomized data (the slow and thorough autopilot mode of PC-ORD). NMS is an iterative search for a ranking that minimizes “stress” of the *k*-dimensional configuration, where stress is a measure of the difference between the distance in the original matrix and the *k*-dimensional configuration. PC-ORD rescales or normalizes stress on a scale of 0–100 using Kruskal’s stress formula 1 [57] and selects the best solution for each dimensionality based on the lowest final stress from a real run. To be considered, the final stress of a dimensionality must be lower than 95% of the randomized runs based on a Monte Carlo test (p<0.05). PC-ORD selects the highest dimensionality (*k*) that reduces stress by 5 or more from the *k*-1 configuration. Solutions with values of 5–10 suggest a good ordination with no real risk of false inference, and values of 10–20 provide a usable overall picture, especially at the lower end. Although NMS is not based on partitioning variance, PC-ORD estimates the proportion of variance represented by an axis from the *r*^2^ between distance in the *k*-dimensional configuration and the original space [56].

## Results

### Mitochondrial DNA

**Fig 4:**
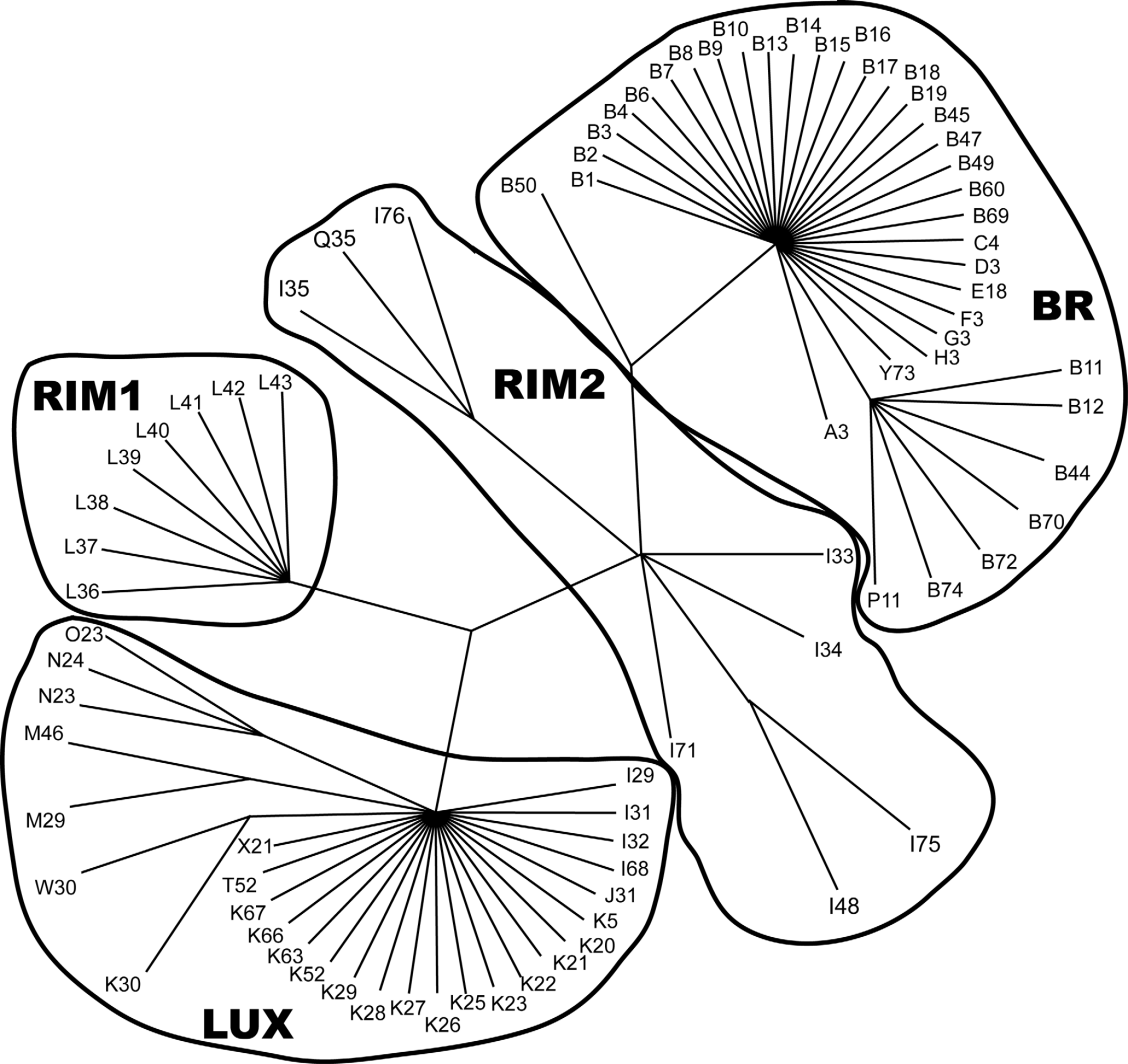
Strict consensus of 1,165,488 most parsimonious trees generated from SSCP haplotype sequences (length = 106 steps, consistency index = 0.55, retention index = 0.93) of Klamath and Rogue basin suckers. Group names as identified in text and haplotypes identified in S2 Table.

SSCP analyses of 665 individuals revealed 21 ND4L, 64 ND2, and 81 composite haplotypes (S2 Table). Parsimony analysis recovered 1,165,488 minimum length unrooted trees (106 steps including uninformative characters, CI = 0.55 [excluding uninformative characters], RI = 0.93). A strict consensus of these trees identified three major clades (Fig 4). One lineage (BR) was composed primarily of morphologically identified *Ch*. *brevirostris* and *C*. *snyderi*, indicating these species could not be separated using mtDNA (S2 Table). Seven *C*. *rimiculus* (all from Lower Klamath River) and five *D*. *luxatus* also had BR haplotypes. The second lineage (LUX) was composed mainly of *D*. *luxatus*, but nine *C*. *snyderi* and 17 *Ch*. *brevirostris* also exhibited LUX haplotypes (S2 Table). The third lineage was composed of *C*. *rimiculus* (S2 Table) from the Rogue River (RIM1), all with ND4L haplotype “L.” The remaining haplotypes were almost exclusively found in *C*. *rimiculus* from the Klamath River and occupied an intermediate position in the consensus tree. This intermediacy was due to homoplasy, as these individuals shared ND4L haplotype “I” with *D*. *luxatus* (discussed below). Given their conspecific classification with Rogue *C*. *rimiculus* based on morphological traits, these individuals are hereafter designated as RIM2. RIM2 haplotypes were also found in two *C*. *snyderi*, one from Klamath River and one from the Sprague River at Chiloquin Dam (S2 Table).

The overall proportion of individuals with mismatched mtDNA (as identified by discordance with morphological identification) was 6.5% (Table 1). *Catostomus rimiculus* exhibited the highest level of mismatch (15.2%), especially below Iron Gate Dam (28.6%) while *Deltistes luxatus* showed the lowest levels of mismatched mtDNA (2.0%). Excluding the Lower Klamath River, Lost River sub-basin (10.2%) showed the greatest amount of mismatch and Upper Williamson River (0%) the least (Table 1).

**Table 1:** Sub-basin percentage of Klamath Basin individuals showing lateral transfer of mtDNA clades (BR, LUX, and RIM2) among field-identified individuals from each of the four species. N is sample size.

Sub-basin	*D*. *luxatus*		*C*. *snyderi*			*C*. *rimiculus*		*Ch*. *brevirostris*		Total %
	N	BR	N	LUX	RIM2	N	BR	N	LUX	
Upper Williamson R.			31							0
Upper Klamath Lake	230	1.7%	124	4.0%	0.8%			61	14.8%	4.6%
Lost River	20	5.0%	38	10.5%				70	11.4%	10.2%
Klamath River										
above Iron Gate			1		100.0%	25	4.0%	14	0.0%	5.0%
below Iron Gate						21	28.6%			28.6%
Total	250	2.0%	194	4.6%	1.0%	46	15.2%	145	11.7%	6.5%

While there was strong correspondence between haplotype lineages and morphology, most individual haplotypes were rare (occurring in fewer than three specimens, **S2 Table**) or, if abundant, found in several species, usually *Ch*. *brevirostris* and *C*. *snyderi* (for example, B3 in 40 *Ch*. *brevirostris*, 64 *C*. *snyderi*, 4 *D*. *luxatus*, and 6 *C*. *rimiculus*). Some haplotypes had unusual geographic distributions relative to patterns of hybridization. For example, LUX haplotype N23 was found in 14 *D*. *luxatus*, all from the Upper Klamath Lake sub-basin. Although clearly part of the *D*. *luxatus* haplotype lineage (Fig 4), N23 was also found in seven *Ch*. *brevirostris* and three *C*. *snyderi*. This was the fourth most common haplotype in Lost River *Ch*. *brevirostris* (10%) and *C*. *snyderi* (7.7%), even though this haplotype was not found in *D*. *luxatus* in this basin. Another high frequency haplotype, F3, was restricted to *C*. *snyderi* from the Upper Williamson River and found in 17 of 31 individuals collected between 14 May and 21 June, 1993. Their ages ranged from 2–20 yrs indicating this was not likely a collection of siblings.

**Fig 5:**
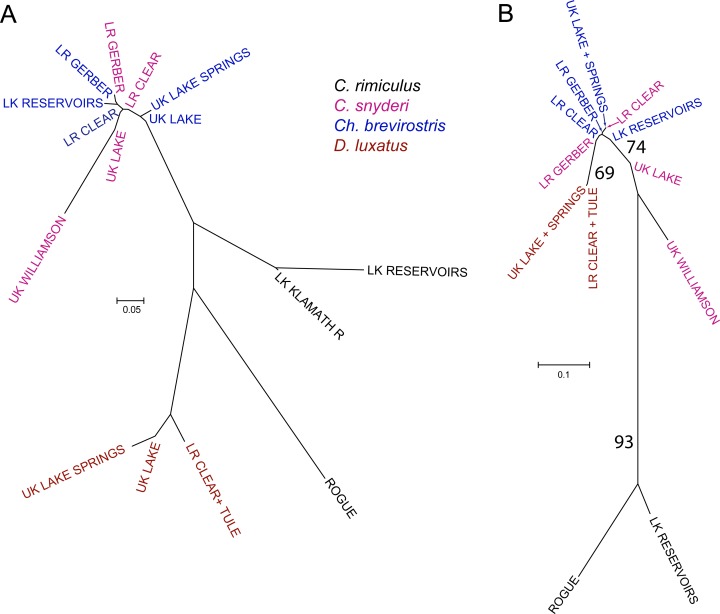
**Population networks of samples from Klamath and Rogue basin suckers based on FST for (A) mtDNA and (B) nuclear genes drawn to scale (provided to the left of each figure).** Colors of labels identify species and LK, LR, and UK are acronyms for Lower Klamath, Lost River, and Upper Klamath, respectively. See Fig 3 for locations.

We quantified the geographic distribution of genetic variation, identifying significant differences among geographic samples (*F*_*ST*_ = 0.66, P < 0.001). When total variance was partitioned by morphological identification, species was found to strongly contribute to divergence, as did geographic location of samples (*F*_*CT*_ = 0.55, P < 0.001; *F*_*SC*_ = 0.24, P < 0.001). Similarity among geographic samples was provided by plotting pairwise *F*_*ST*_ values (Fig 5A), providing results consistent with SSCP parsimony tree. Four lineages were evident, two within *C*. *rimiculus* (Klamath and Rogue rivers), one in *D*. *luxatus*, and a combined lineage that included *Ch*. *brevirostris* and *C*. *snyderi*. It is noteworthy that the sample of *C*. *snyderi* from the Upper Williamson River was distinct from all other samples of that lineage, reflecting the high frequency of a unique haplotype.

**Fig 6:**
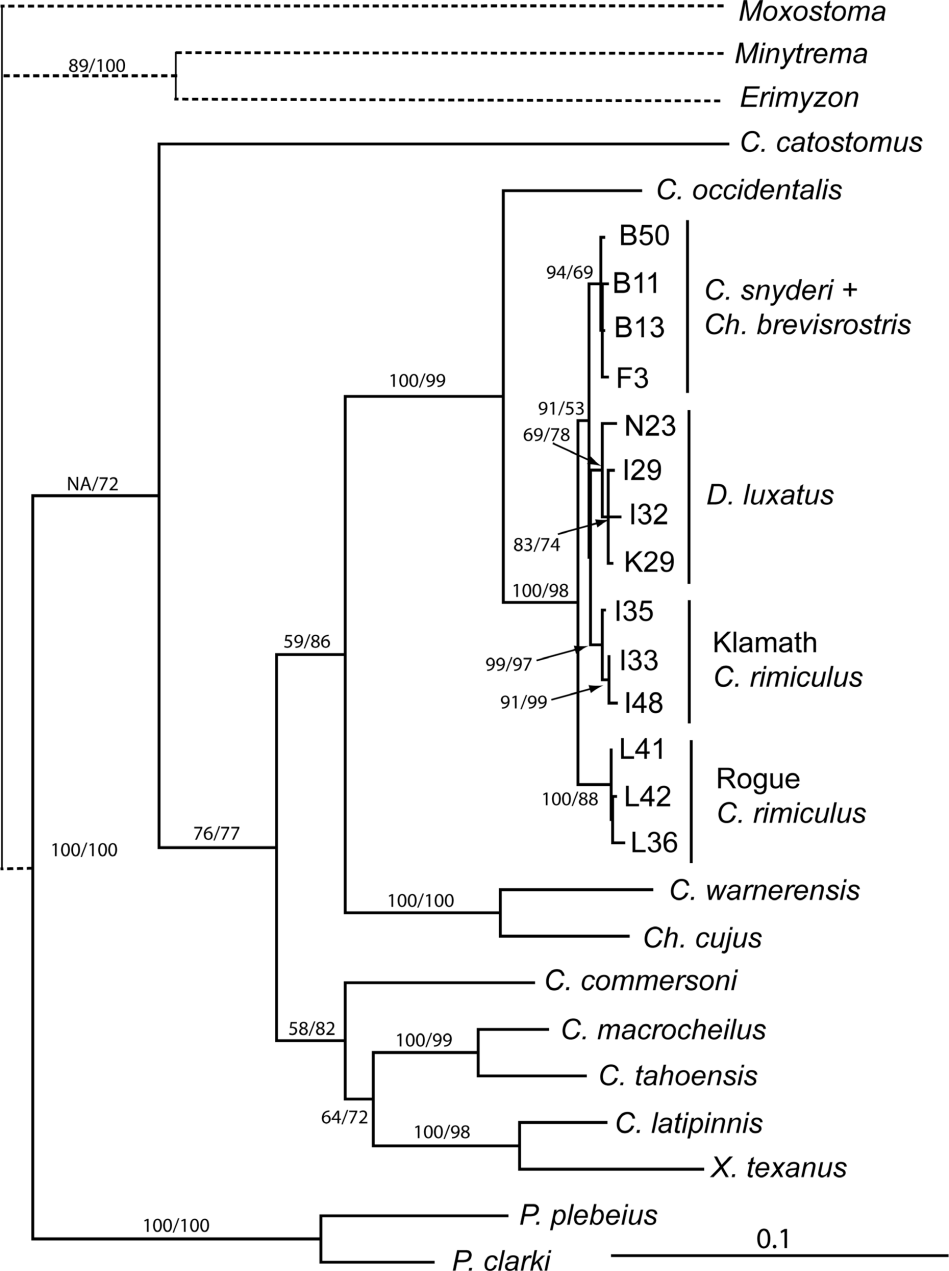
Maximum likelihood tree generated from complete sequences of ND4L, ND2, and cyt*b* genes for a diversity of catostomid fishes. Haplotype labels as in S2 Table. Solid branches are drawn to scale; dashed branches have been truncated to better show details of the ingroup. Numbers near nodes represent bootstrap support (%) for MP and ML analyses, respectively. Values are provided only where one of the values was >70%. NA identifies an inconsistency between MP and ML trees.

Phylogenetic analysis of longer mtDNA sequences of Klamath Basin taxa with representatives of other major catostomin lineages was used to obtain historical perspective of these taxa relative to each other and remaining species of the subfamily. Parsimony analysis recovered 12 trees (length, 2386 [including all characters]; CI = 0.46 [excluding uninformative]; RI = 0.54 –trees not shown), while likelihood analysis recovered a single tree (Fig 6, -Ln likelihood = 13363.943). These topologies and bootstrap re-sampling supported monophyly of each of the four SSCP lineages identified in Fig 4, including RIM2, whose haplotypes were central in the SSCP tree. Note that ND4L haplotype “I” is found in well-supported LUX and RIM2 clades, indicating that the base substitution in the gene responsible for this haplotype is best explained as homoplasious. Haplotypes of the four Klamath Basin suckers formed a well-supported monophyletic group, with Rogue River *C*. *rimiculus* as its sister clade.

To test for consistency between the morphological and mtDNA trees, the sequence data were constrained to maintain monophyly of the *Catostomus* and *Chasmistes* lineages. For this analysis, only sequences from one *Catostomus* (*C*. *occidentalis*) and one *Chasmistes* (*Ch*. *cujus*) were included to reduce the influence of other taxa in the outcome. ML analysis of this subset of taxa without constraints yielded a single topology (-Ln likelihood = 5366.68) that was identical in structure to that obtained from the previous analysis (Fig 6). Because *Ch*. *brevirostris* haplotypes cannot be distinguished from those of *C*. *snyderi*, the analysis was performed twice, with the BR lineage included separately in a monophyletic *Catostomus* and *Chasmistes* lineage, respectively, with both yielding a -Ln likelihood of 5372.21. Therefore, the difference in tree lengths (5.53) is significant [48], indicating that the topology provided by analysis of the morphological traits is not consistent with the one derived from analysis of mtDNA sequences.

### Nuclear markers

Many individuals (range 271–307 individuals, depending on the locus) from the initial set of samples were characterized for variation with nuclear markers. Screening of individuals by restriction endonuclease analysis for Cri1 indicated the restriction site was present in all *C*. *rimiculus* from the Rogue and Klamath basins (allele B), usually as a homozygote (Table 2). It was also found as a heterozygote in two *C*. *rimiculus* from the Klamath River, six *C*. *snyderi* and one *Ch*. *brevirostris*. No *C*. *rimiculus* was homozygous for absence of the site (allele A), and no Upper Klamath Lake or Lost River species was homozygous for presence of the site (Table 2). All samples were in Hardy-Weinberg equilibrium for this locus.

**Table 2:** Number of homozygous and heterozygous individuals of each species for putative nuclear species markers for *C*. *rimiculus*, *C*. *snyderi*, *Ch*. *brevirostris*, and *D*. *luxatus*. Blanks indicate 0 individuals.

Marker	*Ch*. *brevirostris*	*D*. *luxatus*	*C*. *rimiculus*		*C*. *snyderi*		
			Rogue	Klamath	Upper Williamson	Other	Total
Cri1							
AA	112	43			28	27	210
AB	1			2		6	9
BB			30	23			53
Total	113	43	30	25	28	33	272
Cri2							
AA	74	43			28	17	162
AB	30			2	1	11	44
BB	8		30	22		5	65
Total	112	43	30	24	29	33	271
Csn1							
AA	112	46	26	25	9	69	287
AB					18	1	19
BB					1		1
Total	112	46	26	25	28	70	307
L4							
AA	83	42		7	20	57	209
AB	8	1		3	6	9	27
BB	1	1	29	13	1	6	51
Total	92	44	29	23	27	72	287

For locus Cri2, *D*. *luxatus* and Rogue River *C*. *rimiculus* were fixed for alternate alleles (Table 2). Klamath *C*. *rimiculus* was virtually identical to the Rogue form, with only two individuals heterozygous for the allele common to *D*. *luxatus*. *Chasmistes brevirostris* and *C*. *snyderi* were polymorphic, with the *D*. *luxatus* allele more common (ca. 70%). All samples were in Hardy-Weinberg equilibrium for this locus.

For locus Csn1, most individuals were homozygous for allele A (Table 2). Allele B was found as a homozygote in one specimen from the Upper Williamson River and in 18 Upper Williamson River and one Sprague River *C*. *snyderi* that were heterozygous (AB). Twelve of the 19 Upper Williamson River *C*. *snyderi* also had the unique F3 mtDNA haplotype. All samples were in Hardy-Weinberg equilibrium for this locus. Tests for cytonuclear disequilibrium were used to assess distinctiveness of the upper Williamson River population; however, they failed to detect association between this F3 haplotype and nuclear alleles at this locus, but sample sizes may have been too small.

For locus L4, all Rogue River and many Klamath Basin *C*. *rimiculus* were homozygous for the 386 bp fragment (allele B) that was identical or most similar to other western catostomids [44]. The other size variant (397 bp, allele A) was found at high frequency in *C*. *snyderi*, *Ch*. *brevirostris*, and *D*. *luxatus* (Table 2), and only shared with *C*. *occidentalis*, a species found in the Sacramento River of central California [44]. Tests of Hardy-Weinberg equilibrium for polymorphic samples identified deficiencies of heterozygotes in three of the five samples (Klamath *C*. *rimiculus*, *D*. *luxatus*, and *C*. *snyderi* exclusive of the upper Williamson River). Sample sizes and/or rarity of one allele may contribute to these deficiencies. For example, 42 *D*. *luxatus* were AA homozygotes with one homozygote BB male from Clear Lake in Lost River sub-basin and one heterozygous male from Chiloquin Dam in Upper Klamath Lake sub-basin.

We quantified the distribution of genetic variation within and among samples for all four nuclear markers, identifying significant differences within and among them (*F*_*IS*_ = 0.19, P < 0.001; *F*_*ST*_ = 0.63, P < 0.001). The former result reflects deviations from Hardy-Weinberg for some loci in some samples as well as pooling among samples. To investigate factors influencing geographic structure, genetic variation was partitioned by taxonomy as well as location, and species was found to strongly contribute to divergence more strongly than geographic location of samples (*F*_*CT*_ = 0.63, P < 0.017; *F*_*SC*_ = 0.13, P < 0.001). Similarity among geographic samples was provided by plotting pairwise *F*_*ST*_ values (Fig 5B) and by similarity of samples visualized by neighbor joining. Samples of *C*. *rimiculus* and *D*. *luxatus* formed monophyletic groups, supported in 93% and 69% of bootstrap replicates, respectively. The only other group with bootstrap support (74%) included all samples except for *C*. *rimiculus* and the samples of *C*. *snyderi* from Upper Klamath Lake and the upper Williamson River.

### Multicharacter analysis

The original matrix had 252 specimens and 19 coded states (9 morphological and 10 genetic) with no missing data. Eleven outliers were removed from the data set to obtain a reduced matrix. Outliers were either *C*. *snyderi* or *C*. *rimiculus* and included eight of the nine Cri1 heterozygotes in the matrix. These individuals could result from ancestral polymorphism in *Catostomus* or introgression between species. The Cri1 locus was therefore reduced to two states and our final matrix had 241 specimens and 18 coded states. Ordinations with morphological data were rotated to align with gill-raker counts and the genetics-only ordination aligned with the LUX haplotype.

**Fig 7:**
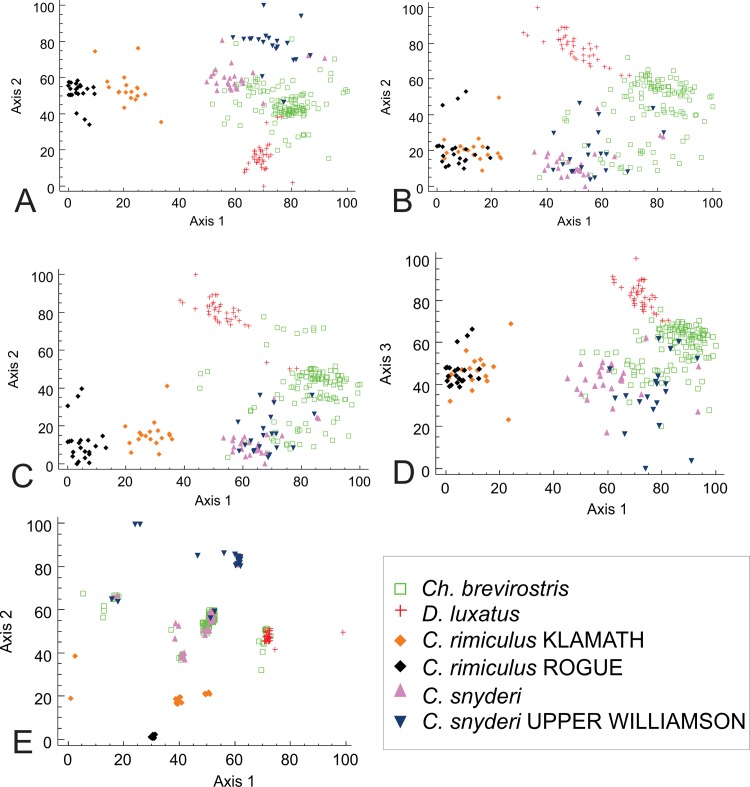
Two major axes for solutions from nonmetric multidimensional scaling of Klamath and Rogue basin suckers. A-D were rotated to align with gillraker counts, E rotated to align with the LUX haplotype. All axes scaled to percentages. A. Two dimensional solution (*k* = 2) using all morphological and genetic characters. B. Two dimensional solution (*k* = 2) using morphological data only. C. Two dimensional solution (*k* = 2) using morphological and mtDNA. D. First and third axes of three dimensional solution (*k* = 3) using morphological and nuclear DNA. E. Two dimensional solution (*k* = 2) using mitochondrial and nuclear data with points jittered to reduce overlap.

To examine the impact of various data sets on the results, several analyses were performed using different combinations of the data (Table 3, Fig 7A–7E), and all yielded either two or three dimensional solutions and were significantly better than chance (Table 3). All p-values were the minimum possible (1/N) based on 250 runs of the Monte Carlo test. All analyses produced low stress and instability values, indicating solutions are stable. Lower stress values suggest less risk in drawing false inferences [56]. The proportions of variance represented by the strongest two axes ranged from 21.0% to 59.3%. The majority of variance was typically explained by the first two axes; however, the comparison using morphology and nuclear characters yielded three significant axes.

**Table 3:** Summary of nonmetric multidimensional scaling analyses for Klamath and Rogue basin suckers. Separate analyses were performed using all data, genetics only, morphology only, morphology plus mitochondrial DNA, and morphology plus nuclear DNA showing the final solution (either two or three dimensional), proportion of variance explained by each axis, final stress, and final instability.

Data	Proportion of Variance			Stress	Instability	Monte Carlo
	Axis 1	Axis 2	Axis 3			
All data	59.3	29.4		12.53	8E-08	0.004
Genetics only	48	40.8		8.26	0.00011	0.004
Morphology only	50.6	41.1		11.68	1E-07	0.004
+ mtDNA	57	36.2		10.24	9E-08	0.004
+ nuclear	58.3	14.1	21	9.51	1E-07	0.004

Nonmetric multidimensional scaling (NMS) of all data had a two dimensional solution (Fig 7A) with proportion of variance represented by the two axes were 39.2% and 49.5%, respectively. This analysis tended to cluster individuals by species; however, there were varying degrees of overlap among all Upper Klamath Basin taxa, with *C*. *rimiculus* completely and *D*. *luxatus* mostly distinct (Fig 7A). In addition, samples of *C*. *rimiculus* from the two basins formed distinct groups, as did *C*. *snyderi* from upper Williamson.

Examination of morphological characters alone by NMS yielded a two dimensional solution with final stress of 11.68, with the first two axes representing 50.6% and 40.1% of the variance, respectively. Rogue and Klamath *C*. *rimiculus* formed a single group distinct from the other species, as did most samples of *D*. *luxatus*. Individuals of *Ch*. *brevirostris* and *C*. *snyderi* were generally distinct; however, there was considerable overlap among these species and considerable scatter of scores for *Ch*. *brevirostris*.

NMS analyses using morphology with mtDNA (Fig 7C) and nuclear characters (Fig 7D) yielded solutions with varying degrees of resolution. The former exhibited a final stress value of 10.24, with the first two axes explaining 57.0% and 36.2% of the variance. In this analysis, the two forms of *C*. *rimiculus* were again distinct, as was *D*. *luxatus*; however, *Ch*. *brevirostris* and *C*. *snyderi* exhibited considerable overlap. When using the morphology and nuclear characters, the final stress value was lower (9.51) and the first and third axes explained most of the variance (58.3% and 21.0%, respectively). This approach again identified *C*. *rimiculus* as distinct but failed to separate the samples from the Rogue and Klamath rivers. Samples of *D*. *luxatus* were mostly distinct; however, some individuals were found in the broadly overlapping cluster of *Ch*. *brevirostris* and *C*. *snyderi*.

The NMS with genetic data only (Fig 7E) had a two dimensional solution with lowest final stress (8.26), with the first two axes explaining 48.0% and 40.8% of the variance, respectively. Species groupings tended to be less distinct than with the total data set; however, Upper Williamson *C*. *snyderi* and Rogue–Klamath *C*. *rimiculus* were again distinct.

## Discussion

The complexity of the Klamath system and analyses of individual characters provided valuable perspective on Klamath Basin taxa and additional insight into the role of hybridization in the evolution of catostomid fishes. Morphological variation had indicated that the four species of suckers in the Klamath and Rogue rivers of Oregon and California were most closely related to other species outside the basin (e.g., *Chasmistes brevirostris* and *D*. *luxatus* with *Ch*. *liorus*, *Ch*. *cujus*, and *Xyrauchen texanus* [27], Fig 1). *Chasmistes*, *Catostomus*, and *Deltistes* are represented in the Miocene fossil record from the Snake River Plain, dating back to 6–9 mA [30]. Phylogenetic and population genetic analyses of mtDNA variation identified samples from the Klamath and Rogue rivers as a divergent lineage that included four distinct groups: *D*. *luxatus*, two forms of *C*. *rimiculus* (from the Klamath and Rogue rivers), and a mixed group of *C*. *snyderi* and *Ch*. *brevirostris*. Samples of *C*. *rimiculus* from the Klamath were more similar to the other species from that basin than to *C*. *rimiculus* from the adjacent Rogue River, a result at odds with analysis of morphological and nuclear traits.

While nuclear markers used here could not be evaluated in a phylogenetic context, Klamath and Rogue river samples of *C*. *rimiculus* shared alleles that were rarely found in the other three taxa and, if so, generally only as heterozygotes (the outliers removed in the NMS), a result consistent with morphological traits. Population genetic analyses were also concordant with expected morphological relationships in that they supported a close relationship of the two populations of *C*. *rimiculus* relative to all other species from the region. *Deltistes luxatus* and upper Williamson River *C*. *snyderi* also were distinct, while *Ch*. *brevirostris* and the remaining samples of *C*. *snyderi* admixed. Analyses of multiple character sets by NMS provided evidence for correlation among character sets, allowing for identification of several morphologically and genetically distinct units, including both samples of *C*. *rimiculus*, upper Williamson *C*. *snyderi*, and *D*. *luxatus*. The major exception was *Ch*. *brevirostris*, which exhibited a broad spread of scores that overlapped with *C*. *snyderi* and, rarely, with *D*. *luxatus*.

While character-based analyses discussed above provided valuable insights into patterns of variation, phylogenetic analyses were critical for examining evolutionary processes. Although broader phylogenetic analyses of mtDNA identified variation consistent with existence of two forms of *C*. *rimiculus* and *D*. *luxatus*, levels of divergence were too low (less than 2%/lineage, Fig 6) given the expected ages of these lineages (6–9 Ma, [30]). In addition, phylogenetic relationships recovered from analysis of mtDNA were consistent with geography (e.g., monophyletic Klamath taxa) and discordant with those provided by analysis of morphological characters, as the mtDNA from the two members of the lake sucker group in the Klamath Basin (*D*. *luxatus* and *Ch*. *brevirostris*) were more closely related to the local *Catostomus* than they were to other *Chasmistes*-like taxa. Examination of the differences between these trees by constraining the mtDNA sequence data to fit the morphological relationships indicated that these discrepancies are statistically significant, identifying conflict between these character sets.

### Interpretation

The strong relationship among character sets and distinct nature of some lineages indicates that each of these forms currently maintains some level of reproductive isolation. The situation for *Ch*. *brevirostris* and *C*. *snyderi* is more difficult to interpret. There is evidence for spatial and temporal segregation during spawning time; however, it was not possible to discriminate among these forms with molecular data.

Search of available data bases for our molecular markers indicated that did not occur in functional genes; therefore, they are presumably neutral. Given this, the most likely explanations for observed results are recent formation of barriers to gene exchange or sufficient leakage of these temporal/spatial barriers to prevent divergence. The antiquity of these species, as demonstrated by the fossil record [30], indicates that barriers to gene exchange should have been in existence as early as 9 million years ago, but the level of mtDNA divergence indicates a relatively recent breakdown in these barriers, likely occurring in the last 3 million years.

The discrepancy between relationships based on mtDNA and morphological characters can be explained by two hypotheses. Introgressive hybridization could have resulted in directional exchange of presumably neutral mtDNA sequence variants among Klamath Basin suckers (making the four species similar) while aspects of the different morphologies are retained through the action of selection. Such patterns of mtDNA introgression appear to be relatively common, especially for mtDNA in fishes [9], including widespread replacement of mtDNA from one species by that of another [58, 59] while the distinct morphological features of lake suckers have been retained for at least 6 my [60].

Alternatively, phylogenetic patterns derived from mtDNA could accurately reflect the evolution of these taxa consequently revealing convergence of morphological traits among lake suckers. In this scenario, morphological similarity could result from convergent evolution of similar adaptive responses to comparable environments, potentially having arisen through sympatric speciation in each lake [17, 61]. If *Chasmistes* are derived from local *Catostomus* in large lakes of western North America through mechanisms like those described by Dieckmann et al. [18], we would expect low levels of molecular divergence across all loci, and apparent monophyly of each pair of *Chasmistes*-*Catostomus* species in each location for neutral molecular markers. The ordination of genetic data (Fig 7) identified a central cluster of *Ch*. *brevirostris* and *C*. *snyderi*, consistent with shared common ancestry of these forms. Monophyly of Klamath Basin taxa and the similarity of mtDNAs among lineages are consistent with shared ancestral polymorphism among lineages, reflecting recent divergence from a common ancestor (Fig 6).

While these features support shared ancestral polymorphism and recent divergence among these taxa, such results are also consistent with past hybridization; therefore, evaluation of these hypotheses requires examination of additional traits. Most important to this issue were the patterns of variation in *C*. *rimiculus*, a form that does not possess trophic specializations found in lake suckers. Although mtDNA lineages from Rogue and Klamath *C*. *rimiculus* did not form a single, monophyletic group, diagnostic morphological characters [31] and fixed (Rogue River) or nearly fixed (Klamath River) diagnostic alleles at three of the four nuclear loci examined (Table 1) support monophyly of this species. In all multi-character analyses, ordination of data showed all *C*. *rimiculus* grouping together separately from Klamath taxa (Fig 6). Analyses of 15 microsatellite loci uncovered similar patterns of variation, with *C*. *rimiculus* and *D*. *luxatus* forming distinct clusters while *Ch*. *brevirostris* and *C*. *snyderi* were indistinguishable [32]. Therefore, nuclear DNA data support monophyly of *C*. *rimiculus* with nuclear heterozygotes in Cri1 (Table 2) indicating ancestral polymorphism or introgression. The nuclear DNA directly conflict with mtDNA, a result most consistent with introgressive hybridization of mtDNA in the evolution of Klamath Basin *C*. *rimiculus*.

Additional information is consistent with these patterns resulting from introgressive hybridization and not convergence of morphological traits. Both *Chasmistes* and *Catostomus* are represented as Pliocene fossils, indicating a long evolutionary persistence of these forms [30]. This trophic polymorphism does not always occur when these taxa invade lakes, as the generalist taxa *C*. *catostomus* and *C*. *commersoni* are often found in lakes without evolution of this morphological specialization; therefore, this trait is not continually arising within the genus as generalist taxa invade lake habitats.

Morphological differences between *Chasmistes* and *Catostomus* are much greater than has been observed in demonstrated ecophenotypes, as many skull and jaw bones are different [60]. These differences are most pronounced in older specimens and erosion of these differences is associated with habitat modification [28]. Therefore, these taxa appear to be in the process of converging, as expected from the impacts of introgressive hybridization.

While there is little doubt that sympatric speciation could happen repeatedly in fishes, there is no plausible ecological or behavioral mechanism to explain how a panmictic ancestral sucker population could develop the spatial and temporal isolation patterns observed in this system. Models of speciation with gene flow generally require life history variation that leads to divergence through reinforcement by assortative mating, such as size-based assortative mating caused by the life history differences [62]. There are differences in maximum size but considerable overlap in sizes of spawners (largely because of their long lives); in addition mating is polyandrous and polygamous. Most importantly, it is unclear as to why a non-specialized form (*C*. *rimiculus*) would re-evolve in both the Klamath and Rogue rivers. Therefore, the weight of the evidence is most consistent with a role for introgressive hybridization among Klamath River suckers, especially evident in *C*. *rimiculus*. With advances in genomics, additional approaches will be helpful in further testing this conclusion.

Patterns of variation described in this paper are not unique and may occur repeatedly in catostomin fishes [28]. Similar patterns have been identified in a related pair of suckers from Utah Lake, June and Utah suckers (*Chasmistes liorus* and *Catostomus ardens*, respectively). Mock et al. [63 identified substantial AFLP and mtDNA sequence differences among populations of *C*. *ardens* from the ancient Snake River drainage and Bonneville Basin; however, Cole et al. [64] used the same characters and found limited differences between *Ch*. *liorus* and *C*. *ardens* in Utah Lake. Microsatellite variation was somewhat consistent with morphological traits, leading the authors to suggest that these patterns could reflect a long history of reticulate evolution. They noted, however, that an alternate explanation could be selection for different morphotypes (benthivorous vs. planktivorous) and recommended additional study to discriminate among these alternatives.

### Implications

Given these considerations, all sucker taxa in the Klamath Basin can be interpreted as a syngameon in which genetic material has moved among each of the species at various times. Unlike other instances of mtDNA introgression, factors responsible for exchange of mtDNA among taxa appear to vary over space and time, as indicated by low levels of divergence among mtDNA lineages and distinctiveness of these taxa as indicated by population genetic and morphological analyses. Klamath River *C*. *rimiculus* from above Iron Gate Dam rarely exhibit the affects of introgression of the BR clade (4%), yet the BR clade is common (28.5%) in *C*. *rimiculus* taken below the dam (Tables 2 and 3). Molecular evidence indicates that introgression into *D*. *luxatus* is also limited as 98% of individuals representing this taxon exhibited LUX mtDNA haplotypes and were fixed (Cri1, Cri2 and Csn1) or nearly fixed (L4) for Klamath Basin nuclear alleles (Tables 1 and 2). Reciprocal transfer of LUX haplotypes to *C*. *snyderi* and *Ch*. *brevirostris* was more frequent but still uncommon (4–14.8%, Table 1). This is somewhat surprising as recent radio-tagging of Sprague River spawning fish by USGS showed *D*. *luxatus* was more likely to overlap spatially and temporally with both *C*. *snyderi* and *Ch*. *brevirostris* than either of those two with each other, providing greater opportunity for hybridization (Table 4). Since *D*. *luxatus* is also the largest sucker in the basin, its large females may attract males from other species during spawning overlap and account for the asymmetry in mtDNA transmission. Confirmation of this hypothesis requires additional nuclear markers for discriminating all species.

**Table 4:** Approximate spawning times and locations for suckers in the Sprague River based on USGS radio tagged specimens in 2005. Rkm = approximate distance from Upper Klamath Lake in river km. K = *C*. *snyderi*, L = *Deltistes luxatus* and S = *Ch*. *brevirostris*). Early and late refer to the first two or last two weeks of the month. Data from Ellsworth and Shively, USGS.

		March		April		May	
Reach	Rkm	early	late	early	late	early	late
North Fork Sprague	~145				K		
Sycan River	~140				K		
Beatty Gap	129–137		K	KL	KL		
Nine Mile	30–63		K	KL	L		
Below Chiloquin Narrows	10–27			L	LS	LS	S

Given our inability to discriminate between *C*. *snyderi* and *Ch*. *brevirostis* with molecular markers, these taxa are currently or have recently been hybridizing. All differences in these species were geographic and inconsistent with morphology [32]. Within *C*. *snyderi*, only Upper Williamson fish had evidence of isolation based on both morphological (relatively large head [30]) and molecular data (Fig 7). The molecular evidence for distinctiveness of this group included a high frequency (36%) of allele B at locus Csn1 (Table 2), high frequency (>60%) of a unique mtDNA haplotype, F3, and microsatellite differences [32].

Within Upper Klamath Lake sub-basin, *Ch*. *brevirostris* and *C*. *snyderi* are morphologically diagnosable [31] and spawning times and locations differ, with *Ch*. *brevirostris* spawning later in near-shore springs in the lake (now rare) and in the lower 30 km of the Williamson and Sprague rivers, while *C*. *snyderi* spawns earlier in the upper 30–150 km of the rivers (Table 3) and has never been detected spawning in the lake. If hybridization is responsible for these patterns, lateral transfer might be mediated either through river-spawning *D*. *luxatus*, through overlap in spawning time with both species, or through historical habitat/climate changes. There was concern locally that recent introgression might be partly due to the former Chiloquin Dam on the Sprague River, which impeded upriver migrants since 1914 and may have partly broken down spatial segregation among river spawners. However, the BR haplotype clade is also found in lake-spawning *Ch*. *brevirostris* and Upper Williamson *C*. *snyderi*, so its presence in both species likely predates the dam.

Although the underlying genetic basis may differ, ecological conditions seem to be most important for maintaining species identity in adaptive speciation and syngameons [6]. In the Klamath Basin, those conditions apparently differ in sub-basins. For example, downstream Klamath River reservoirs also contain *Ch*. *brevirostris* that are derived from Upper Klamath Lake [32], but reservoir flushing rates are so great that any larvae produced are not retained. We believe *Ch*. *brevirostris* from Upper Klamath Lake sub-basin are the source for populations in Klamath River, as ecological conditions are not sufficient to maintain the species. The result is a small population in Klamath River that is unable to sustain itself.

## Conclusions

Morphological and molecular evidence presented here, along with the fossil record, indicates that Klamath Basin suckers belong to reticulate lineages that over time have joined, separated, and essentially formed a basin-wide syngameon. Grant [65] considers a species in a syngameon as “the most inclusive unit of interbreeding in a hybridizing species group” and Seehausen [2] as “a complex of selection-maintained, genetically weakly but ecologically highly distinctive species capable of exchanging genetic material.” Mayr [66] argued against hybrids as a source of genetic variation available for positive effects on fitness of species, noting that the cost in wasted gametes would be high and the benefits would be outweighed by the disruption of co-evolved gene complexes. He also noted that fish differed from most land vertebrates in the higher frequency of hybrids, which he attributed to external fertilization “among other” reasons. The most important source of demographic difference between teleosts and other vertebrates is their small eggs, high fecundity, and 90+% mortality of eggs and larvae [67,68]. The long life and high fecundity of Klamath Basin suckers (up to 57 yr. and 235,000 eggs/yr. in *D*. *luxatus* [69–72]) may make the cost of wasted gametes trivial. The tetraploid genome, on the other hand, may allow for retention of unaltered copies of important, co-evolved gene complexes and facilitate existence both of the syngameon and its constituent species. There are many examples of plant syngameons, such as balsam poplar and cottonwoods, which have been ecologically and evolutionary distinct for 12 million years and hybridizing throughout [6]. Often these hybridizing species do not show differences at most molecular markers but do show morphological and ecological differences and sometimes differences in rapidly evolving microsatellite DNA [73].

Since Klamath suckers appear to be part of a syngameon in which neutral and possibly advantageous loci are moving, and have previously moved, between species, phylogenetic relationships obtained from molecular data are obscured due to the existence of multiple, reticulate evolutionary histories [7, 14, 74]. The cohesion of species in syngameons is maintained by selection [75]. Because members of the syngameon would share neutral and adaptive variants, each member of the unit would exhibit increased effective population sizes, increasing variation in the complex, and presumably overall levels of adaptation [3, 76, 77]. This cohesion also confounds our ability to distinguish taxa with molecular markers [78]. Conflicts in phylogenetic signal are most likely to involve mtDNA because of its strictly maternal inheritance and rapid rate of evolution, a prediction that has been supported numerous times for fishes [79–82]. Under this scenario, phylogenetic patterns (such as the apparent para- or polyphyly of *C*. *rimiculus*) and low levels of divergence among Klamath taxa reflect the influence of cycles of past hybridization.

This behavior has significant and far-reaching consequences relative to recovering phylogenetic relationships. If our data reflect general processes, then a large part of genome may be continuously impacted by introgression. The demonstration of “speciation” genes [78, 83–84] suggests a mechanism whereby a small part of the genome is protected and its phylogeny alone is congruent with traits used to define the species. Given that other traits will evolve independent of such genes, an intractable bias is introduced, detracting from our ability to infer accurate phylogenetic relationships of such organisms [7, 23, 78]. Temporal variability in patterns and levels of introgression will significantly complicate matters because this process could produce apparent but false resolution of the evolutionary history of the lineage due to divergence following introgression.

In systems where introgressive hybridization is frequent, we need to adjust our expectations relative to understanding phylogenetic relationships. In these systems, evolutionary history cannot be viewed in the typical conventional form of a tree with distinct branches that can represent estimated relationships. A more appropriate approach would be to realize that different blocks of the genome will have distinct evolutionary histories, determined by the specific evolutionary processes driving them. If this is correct, there is more than one history for these organisms, and evolutionary patterns would best be reflected as a network, as suggested by Beiko et al. [74] for bacteria.

Finally, the existence of a syngameon has important implications for endangered species management and for systematic research. In the Klamath Basin, questions of protecting unlisted but similar species (*C*. *snyderi*) or of controlling hybrids have different answers depending upon the interpretation of the system. Furthermore, a taxon with one population participating in a syngameon, while another does not, leads to questions about evolutionary independence and identification of “species.” Upper Williamson River *C*. *snyderi* and Rogue River *C*. *rimiculus* deserve further study in this regard. In addition, if much of the genetic diversity in Klamath Basin suckers is potentially available to all species, both the unlisted and listed species are part of a system that allows for quick increases in variability and rapid response to change [1]. If this is a correct description of the system, the conservation of all of its elements, including hybridization dynamics, is important.

## Supporting Information

S1 Table Locality data and morphological and genetic data for each trait and individual examined in this study of catostomid fishes from the Klamath and Rogue rivers. (XLSX)

S2 Table Number of individuals with different SSCP haplotypes in each of the different species, including both drainages represented by *C*. *rimiculus*. Haplotypes and mtDNA clades are identified in Fig 4.
(XLSX)
